# Variations in Processes of Care and Outcomes for Hospitalized General Medicine Patients Treated by Female vs Male Physicians

**DOI:** 10.1001/jamahealthforum.2021.1615

**Published:** 2021-07-16

**Authors:** Anjali Sergeant, Sudipta Saha, Saeha Shin, Adina Weinerman, Janice L. Kwan, Lauren Lapointe-Shaw, Terence Tang, Gillian Hawker, Paula A. Rochon, Amol A. Verma, Fahad Razak

**Affiliations:** 1 McMaster University, Hamilton, Ontario, Canada; 2 St Michael’s Hospital, Toronto, Ontario, Canada; 3 Unity Health Toronto, Toronto, Ontario, Canada; 4 Sunnybrook Health Sciences Centre, Toronto, Ontario, Canada; 5 University of Toronto, Ontario, Canada; 6 Women’s College Hospital, Ontario, Canada

## Abstract

**Importance:**

Hospitalized medical patients cared for by female physicians may have decreased mortality rates compared with patients of male physicians. However, this association has yet to be assessed outside of the US, and little is known about factors that may explain this difference.

**Objective:**

To determine whether mortality, other hospital outcomes, and processes of care differed between the patients cared for by female and male physicians.

**Design, Setting, and Participants:**

This retrospective cross-sectional study included patients admitted to general medical wards at 7 hospitals in Ontario, Canada, between April 1, 2010, and October 31, 2017. The association of physician gender with patient outcomes was examined while adjusting for hospital fixed effects, patient characteristics, physician characteristics, and processes of care. All patients were admitted to a general internal medicine service through the emergency department and were cared for by a general internist or family physician-hospitalist. Patients were excluded if length of stay was greater than 30 days or if the attending physician cared for less than 100 hospitalized general medicine patients over the study period. Statistical analyses were performed from October 15, 2020, to May 8, 2021.

**Main Outcomes and Measures:**

In-hospital mortality, length of stay, intensive care unit admission, 30-day readmissions, and process-of-care measures (blood tests, medications, imaging, endoscopy, and interventional radiology services).

**Results:**

A total of 171 625 hospitalized patients with a median age of 73 years (interquartile range, 56-84 years) were included (84 221 men [49.1%], 87 402 women [50.9%], and 2 patients with unspecified sex). Patients were cared for by 172 attending physicians (54 female physicians [31.4%] and 118 male physicians [68.6%]). In fully adjusted models, female physicians ordered more imaging tests, including computed tomography (adjusted difference, −1.70%; 95% CI, −2.78% to −0.61%; *P* = .002), magnetic resonance imaging (−0.88%; 95% CI, −1.37% to −0.38%; *P* = .001), and ultrasonography (−1.90%; 95% CI, −3.21% to −0.59%; *P* = .005). Patients treated by female physicians had lower in-hospital mortality (2256 of 46 772 patients [4.8%] vs 6452 of 124 853 patients [5.2%]). This difference persisted after adjustment for patient characteristics but was no longer statistically different after adjustment for other physician characteristics (adjusted difference, 0.29%; 95% CI, −0.08% to 0.65%; *P* = .12). The difference was similar after further adjustment for processes of care.

**Conclusions and Relevance:**

In this cross-sectional study of patients admitted to general medical units in Canada, patients cared for by female physicians had lower mortality rates than those treated by male physicians, adjusting for patient characteristics. This finding was nonsignificant after adjustment for other physician characteristics.

## Key Points

**Question:**

Is physician gender associated with mortality and other patient outcomes in a general internal medicine inpatient setting?

**Findings:**

In this cross-sectional study of 171 625 hospitalized patients, patients cared for by female physicians had lower in-hospital mortality after adjustment for hospital and for patient characteristics, but this was no longer statistically different after adjustment for physician characteristics.

**Meaning:**

The lower mortality rate in patients cared for by female physicians may be partially explained by differences in physician characteristics.

## Teaser

This cross-sectional study investigates the association of physician gender with mortality and patient outcomes in a general medicine inpatient setting.

## Introduction

A 2017 study of patients admitted to internal medicine wards in the US noted that those cared for by female physicians had a lower 30-day mortality rate.^1^ This finding added to prior evidence, largely from primary care settings, of differences in practice patterns between female and male physicians. Specifically, female physicians are more likely to provide preventive care,^2,3,4,5,6,7,8,9^ adhere to clinical guidelines,^10,11,12,13^ take a patient-centered approach,^14,15,16^ perform better on qualifying examinations,^17^ and spend more time in direct patient care for lower remuneration.^18^ However, to our knowledge, the difference in patient mortality between female and male physicians has not been evaluated outside of the US, and little is known about what factors may be contributing to this difference in outcomes.

There is a dearth of research examining sex and gender differences in processes of care, which are defined as “technical interventions and interpersonal interactions between users and members of a healthcare system”^19^^(p1613)^ and include the physician’s diagnostic and therapeutic actions.^20^ Our study seeks to improve the understanding of differences between male and female physicians in the processes of care and patient outcomes. We hypothesize that female physicians perform more diagnostic tests than male physicians, which may explain a lower patient mortality rate.

We examined inpatients from the General Medicine Inpatient Initiative (GEMINI) retrospective cohort, which involves 7 hospitals in the greater Toronto area.^21,22^ Our study objectives were to (1) examine differences in blood tests, imaging tests, and medications ordered by male and female physicians; (2) determine whether female and male physicians have differences in major patient outcomes, including mortality; and (3) assess whether patient characteristics, physician characteristics (eg, specialty, years of experience), or processes of care explain any observed differences in outcomes.

## Methods

### Design and Setting

This was a cross-sectional study of patients from the GEMINI cohort, a multicenter retrospective study that includes patient data from hospital sites associated with the University of Toronto in Ontario, Canada, from April 1, 2010, to October 31, 2017. The participating organizations are St Michael’s Hospital, Sinai Health System (Mount Sinai Hospital), Sunnybrook Health Sciences Centre, Trillium Health Partners (Credit Valley and Mississauga hospitals), and the University Health Network (Toronto General Hospital and Toronto Western Hospital). These organizations are independent care providers with distinct governance and health records.^21^ Ethics approval was obtained from the research ethics boards at all participating hospitals before the collection of retrospective data. Participant consent was waived as the data were deidentified before use. This study was conducted in accordance with the Strengthening the Reporting of Observational Studies in Epidemiology (STROBE) reporting guideline for cross-sectional studies.^23^

### Inclusion and Exclusion Criteria

Our study included patients who were admitted to or discharged from a general medical service from April 1, 2010, to October 31, 2017.^21^ Only patients admitted to the general internal medicine (GIM) ward via the emergency department were included; we excluded patients admitted from any other source to avoid elective admissions or interhospital transfers for which physician assignment may be nonrandom. For emergency department admissions, the Canadian Institute of Health Information (CIHI) database retrospectively assigns admitted patients to a most responsible physician (MRP). The MRP is defined as the attending physician who is most “responsible for the patient’s care or who cared for the patient the longest” during their hospital stay,^24^^(p17)^ and this definition is widely applied in epidemiological studies.^25,26,27,28^ We excluded hospitalizations if the MRP’s gender was not recorded or if patient length of stay (LOS) in the hospital was more than 30 days (as longer stays often lead to multiple physician handoffs).^22^ Physicians who cared for fewer than 100 patients over the study period in the GIM ward and patients admitted to nongeneralist specialty wards that existed at some hospitals (eg, poststroke care ward) were also excluded.

### Data Collection

As described in detail elsewhere,^21,29^ patient data from hospital administrative sources were collected from hospitals as reported to the CIHI Discharge Abstract Database. Data extracted from electronic health records included laboratory tests, medical imaging, and in-hospital medication orders and were linked to hospital administrative data.

All data pertaining to physician characteristics, including physician gender, years of practice, medical school, and specialty, were collected from the publicly accessible College of Physicians and Surgeons of Ontario (CPSO) website.^30^ Sex and gender are distinct, but often overlapping, identifiers.^31^ We use the term *gender* in reference to physicians because the CPSO categorizes gender as male or female based on physician self-classification upon application for a medical license.^32^

### Measures

Baseline patient characteristics in descriptive statistics and multivariable adjustment include patient age at admission (linear in models), patient sex, time of admission (weekday vs weekend and daytime vs nighttime), fiscal year of admission (linear), and admitting hospital (categorical). We included the patient’s most responsible diagnosis, grouped into major disease categories based on the Clinical Classifications Software.^33^ We measured comorbidity using the Charlson Comorbidity Index score (0, 1, 2+), where higher numerical scores estimate a decreased 10-year survival rate.^34^ Severity of illness was assessed using the Laboratory-Based Acute Physiology Score (linear), a validated predictor of inpatient mortality.^35,36^ We also included prior admission to 1 of the 7 GIM hospital sites in the previous 30 days. Baseline characteristics were compared using standardized mean differences (mean difference between female and male physicians, divided by the SD across all admissions), where values greater than 0.1 are considered meaningful markers of imbalance.^37^ A multivariate Mahalanobis distance-based method was used for multinomial outcomes.^38^

Physician characteristics included in descriptive statistics and multivariable analysis were physician gender, physician specialty (family medicine vs internal medicine), years of experience (defined as years in independent medical practice at time of encounter and modeled linearly), and graduating medical school location (categorized as Canadian, US, or international).

### Outcomes

The primary outcome was in-hospital patient mortality; secondary outcomes included intensive care unit admission, hospital LOS, cost of care, and readmission to GIM at 1 of the GEMINI hospitals within 30 days of discharge. Processes of care can encompass a wide range of physician activity^39,40^; we included commonly ordered laboratory tests,^21^ imaging,^21^ and medications^41,42^ available in electronic medical records. These variables included routine blood tests ordered per patient day; acute blood tests ordered; imaging tests (ie, x-ray, computed tomography [CT] scan, magnetic resonance imaging [MRI], and ultrasound), interventional radiology procedures; in-hospital endoscopy; transfusions; and select medications ordered in the hospital (ie, antimicrobials, anticoagulants, benzodiazepines, and antipsychotics) that carry a substantial risk of adverse events in a GIM population.^43,44,45^ A detailed description of the medications included is available in eTable 10 in the Supplement. Data on blood transfusions were derived from blood bank orders or CIHI-reported fields, depending on availability by site.

To estimate costs of hospitalization across study sites and years, we used the CIHI Resource Intensity Weight^46^ for each patient admitted and multiplied this value by the annual cost per weighted case using the Ontario Cost Distribution Methodology.^47^ By accounting for patient age, comorbidities, and diagnosis at discharge, this method provides an estimation in Canadian dollars of the average amount of hospital resources used for each hospitalization but does not include fee-for-service physician billing.^48^

### Statistical Analysis

A key assumption applied in this analysis was that nonelective admissions from the emergency department to internal medicine wards were assigned in a quasirandomized process to the physician who was on call for general medical admissions. This process implies that patient characteristics should be balanced between female and male physicians within each hospital at the start of the admission process and that any differences in processes of care and clinical outcomes may then be related to differences in physician practice.^22^ This assumption was an underlying principle applied in the study by Tsugawa and colleagues,^1^ and it has been tested rigorously in GEMINI.^21,22^

Following the approach used by Tsugawa and colleagues,^1^ we used generalized linear models to estimate the association of physician gender with patient outcomes and processes of care with SEs adjusted for clustering of patients within physicians. We used logistic regression for binary outcomes (in-hospital death, intensive care unit admission, 30-day readmission, use of advanced imaging, endoscopy, interventional radiology, and transfusion and medication orders); linear regression for log-transformed in-hospital costs; and negative binomial regression for LOS and routine blood tests per day (with an offset for LOS). A staged multivariable modeling approach was used. Model 1 included physician gender and hospital fixed effects. Hospital fixed effects enable an effective comparison of female and male physicians practicing within different hospitals.^1^ Model 2 added patient factors, model 3 added physician characteristics, and model 4 added processes of care. Additionally, we examined the effect of adjusting for processes of care without physician characteristics included. For differences of interest, marginal standardization was used to estimate adjusted prevalence of outcome and process variables by physician gender.^49^ We tested for collinearity by calculating generalized variance inflation factors, which were fewer than 2 for all variables in the fully specified models.

Multiple sensitivity analyses were performed to assess the robustness of our findings. First, a more restrictive cohort was used in which the same physician was the MRP, the admitting physician, and discharging physician. We used this model to increase the likelihood that a patient was treated by the same physician across their entire stay. A second sensitivity analysis excluded patients receiving palliative care as defined by the *International Statistical Classification of Diseases and Related Health Problems, Tenth Revision Canada* code Z515. Third, we examined the differences in in-hospital mortality among cohorts including only female patients or only male patients in order to investigate whether the physician gender association differed by the sex of the patient. Fourth, we included models adjusting for hospital, patient characteristics, and physician years of experience to evaluate whether years of experience was sufficient to attenuate mortality differences. Fifth, we repeated the models of the main analysis with physician years of experience as a categorical variable and also as a linear variable with quadratic and cubic terms. Statistical analyses were performed from October 15, 2020, to May 8, 2021, using R software, version 4.0.2 (R Core Team). All *P* values were 2-sided, and *P <* .05 was considered significant.

## Results

From an initial 228 450 hospitalizations in the GEMINI database, 171 625 hospitalized patients cared for by 172 MRPs were included in this study on the basis of the inclusion and exclusion criteria (Figure). The median patient age was 73 years (interquartile range [IQR], 56-84 years); 84 221 (49.1%) were men, 87 402 (50.9%) were women, and 2 had no sex specified. The proportions of female and male physicians in the study (54 female physicians [31.4%] and 118 male physicians [68.6%]) were not significantly affected by any of the inclusion or exclusion steps. The proportion of female physicians at each hospital ranged from 23% to 38%. The characteristics of the 54 female and 118 male physicians are presented in Table 1. Median duration in practice was 4.3 years (IQR, 2.5-11.5 years) for female physicians and 7.4 years (IQR, 3.3-16.4 years) for male physicians. No significant differences were noted between female and male physicians in location of medical school training, specialty, or hospital of practice. Patient characteristics were largely balanced between female and male physicians, with standardized differences less than 0.3 for all characteristics (Table 2). Fiscal year of admission showed an increased proportion of patients attributed to female physicians in more recent years (eTable 1 in the Supplement).

**Figure.**
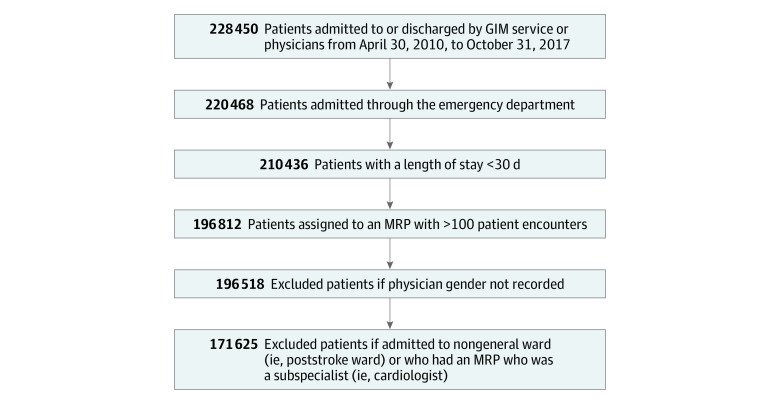
Inclusion and Exclusion Step Flowchart GIM indicates general internal medicine; MRP, most responsible physician.

**Table 1.** Characteristics of Female and Male Physicians

Physician characteristic	Physician, No. (%)	
	Female (n = 54)	Male (n = 118)
Years of experience, median (IQR)	4.3 (2.5-11.5)	7.4 (3.3-16.4)
Medical school education		
Canadian medical school	48 (88.9)	99 (83.9)
US medical school	3 (5.6)	14 (11.9)
International medical school	3 (5.6)	5 (4.2)
Internal medicine specialty	46 (85.2)	107 (90.7)

Abbreviation: IQR, interquartile range.

**Table 2.** Patient Characteristics by Physician Gender

Patient characteristic	Patients, No. (%)		Standardized mean difference
	Cared for by female physician (n = 46 772)	Cared for by male physician (n = 124 853)	
Age, median (IQR)	73 (57-84)	73 (56-84)	.02
Sex			
Female	24 050 (51.4)	63 352 (50.7)	.01
Male	22 721 (48.6)	61 500 (49.3)	
Other^a^	<5	<5	NA
Charlson Comorbidity Score 2+	19 911 (42.6)	52 748 (42.2)	.01
Laboratory Acute Physiology Scale, median (IQR)	17.0 (6.0-29.0)	17.0 (6.0-30.0)	.01
Time of admission: nighttime	36 553 (78.2)	97 264 (77.9)	.01
Day of admission: weekend	12 065 (25.8)	32 783 (26.3)	.02
Previous admission to GIM hospital in past 30 d	5054 (10.8)	13 717 (11.0)	.01
Principal diagnosis at discharge			
Chronic obstructive pulmonary disease	1981 (4.2)	5570 (4.5)	.03
Delirium, dementia, cognitive disorders	1561 (3.3)	3950 (3.2)	
Fluid and electrolyte disorders	1213 (2.6)	3240 (2.6)	
Gastrointestinal hemorrhage	1217 (2.6)	3445 (2.8)	
Heart failure	2201 (4.7)	6054 (4.8)	
Intestinal infection	1183 (2.5)	3136 (2.5)	
Other	29 734 (63.6)	79 615 (63.8)	
Pneumonia	2574 (5.5)	6607 (5.3)	
Septicemia (excluding during labor)	1137 (2.4)	3105 (2.5)	
Stroke	1439 (3.1)	3591 (2.9)	
Urinary tract infection	2532 (5.4)	6540 (5.2)	

Abbreviations: GIM, general internal medicine; IQR, interquartile range; NA, not applicable.

^a^ Other refers to patients for whom data were not collected.

Table 3 depicts the differences in hospital outcomes by physician gender. The in-hospital mortality rate was lower among patients treated by female physicians compared with those cared for by male physicians (unadjusted rates, 2256 of 46 772 [4.8%] vs 6452 of 124 853 [5.2%]). The mortality difference persisted after adjustment for hospital fixed effects (adjusted odds ratio [AOR], 1.11; 95% CI, 1.01-1.23; *P* = .04) and patient baseline characteristics (AOR, 1.12; 95% CI, 1.01-1.24; *P* = .03). The adjusted mortality was 4.7% for patients of female physicians and 5.2% for those of male physicians (risk difference [RD], 0.47%; 95% CI, 0.03%-0.9%; *P* = .03). This difference was no longer significant after adjustment for physician characteristics (RD, 0.29%; 95% CI, –0.08% to 0.65%; AOR, 1.07; 95% CI, 0.99-1.17; *P* = .12). The estimate was not further attenuated after adjustment for processes of care (AOR, 1.07; 95% CI, 0.99-1.17; *P* = .10). Additionally, without adjusting for physician characteristics, adjusting for processes of care did not attenuate the mortality difference (AOR, 1.13; 95% CI, 1.03-1.24; *P* = .01) (eTable 2 in the Supplement). Patients of female physicians had a higher median cost per admission ($4694.50; IQR, $2587.60-$8727.10 vs $4386.90; IQR, $2390.00-$8305.30), which persisted in all adjusted models (fully adjusted effect, −3.44%; 95% CI, −5.08% to −1.77%; *P* < .001). Although the unadjusted median LOS was identical for female and male physicians, patients of female physicians had higher LOS in adjusted models (fully adjusted rate ratio, 0.98; 95% CI, 0.96-0.99; *P* = .006). Differences in intensive care unit admission and 30-day readmission rates were not significant.

**Table 3.** Association Between Physician Gender and Patient Outcomes

Outcome	No. (%) of patients or median (IQR)		Model 1^a^ (hospital effects)		Model 2 (+ patient characteristics)		Model 3 (+ physician characteristics)		Model 4 (+ processes of care)	
	Cared for by female physician (n = 46 772)	Cared for by male physician (n = 124 853)	OR/RR/effect (95% CI)	*P* value	OR/RR/effect (95% CI)	*P* value	OR/RR/effect (95% CI)	*P* value	OR/RR/effect (95% CI)	*P* value
Mortality^b^	2256 (4.8)	6452 (5.2)	1.11(1.01 to 1.23)	.04	1.12 (1.01 to 1.24)	.03	1.07 (0.98 to 1.17)	.12	1.07 (0.99 to 1.17)	.10
30-d Readmission^b^	5327 (12.1)	14 557 (12.4)	1.01 (0.97 to 1.05)	.64	1.02 (0.98 to 1.06)	.39	1.02 (0.98 to 1.06)	.24	1.03 (0.99 to 1.07)	.22
ICU admission^b^	2300 (4.9)	6207 (5.0)	0.96 (0.88 to 1.04)	.33	0.96 (0.89 to 1.05)	.38	0.93 (0.86 to 1.00)	.06	0.95 (0.88 to 1.04)	.26
Length of stay^c^	5.0 (3.0 to 9.0)	5.0 (3.0 to 8.0)	0.97 (0.94 to 1.00)	.06	0.97 (0.95 to 1.00)	.03	0.97 (0.94 to 0.99)	.01	0.98 (0.96 to 0.99)	.006
Total cost, $^d^^,^^e^	4694.50 (2587.60 to 8727.10)	4386.90 (2390.00 to 8305.30)	−5.00 (−8.15 to −1.74)	.003	−4.96 (−7.71 to −2.13)	.001	−5.28 (−7.90 to −2.59)	<.001	−3.44 (−5.08 to −1.77)	<.001

Abbreviations: ICU, intensive care unit; IQR, interquartile range; OR, odds ratio; RR, rate ratio.

^a^ Model 1, hospital effects; Model 2, hospital effects plus patient characteristics; Model 3, hospital effects plus patient characteristics and physician characteristics; Model 4, hospital effects plus patient characteristics and physician characteristics and processes of care.

^b^ Odds ratio from logistic regression.

^c^ Rate ratio (days per admission) from negative binomial regression.

^d^ Effect size on log of total cost expressed as percentage change.

^e^ Units are in Canadian dollars.

Processes of care are presented in Table 4. Female physicians ordered more imaging tests than male physicians (CT, 25 615 of 46 772 patients [54.8%] vs 64 868 of 124 853 patients [52.0%]; MRI, 5202 patients [11.1%] vs 12 688 patients [10.2%]; and ultrasound, 14 832 patients [31.7%] vs 36 195 patients [29.0%]). For CT, MRI, and ultrasound, this difference persisted in all adjusted models (CT AOR, 0.93; 95% CI, 0.89-0.97; *P* = .002; MRI AOR, 0.90; 95% CI, 0.85-0.96; *P* = .001; and ultrasound AOR, 0.91; 95% CI, 0.85-0.97; *P* = .005). The fully adjusted order rate for CT scans was 54.0% and 52.3% for female and male physicians, respectively (RD, −1.70%; 95% CI, −2.78% to −0.61%). For MRI, the adjusted rates were 11.1% vs 10.2% (RD, −0.88%; 95% CI, −1.37% to −0.38%]). For ultrasound, the adjusted rates were 31.1% vs 29.2% (RD, −1.90%; 95% CI, −3.21% to −0.59%). The use of x-ray, endoscopy procedures, interventional radiology procedures, blood transfusion, routine or acute blood tests, and medication orders did not differ significantly.

**Table 4.** Association Between Physician Gender and Processes of Care

Process of care	Patients, No. (%)		Model 1 (hospital effects)		Model 2 (+ patient characteristics)		Model 3 (+ physician characteristics)	
	Cared for by female physician (n = 46 772)	Cared for by male physician (n = 124 853)	OR/RR/effect (95% CI)	*P* value	OR/RR/effect (95% CI)	*P* value	OR/RR/effect (95% CI)	*P* value
CT imaging^a^	25 615 (54.8)	64 868 (52.0)	0.91 (0.87 to 0.95)	<.001	0.91 (0.87 to 0.95)	<.001	0.93 (0.89 to 0.97)	.002
MRI imaging^a^	5202 (11.1)	12 688 (10.2)	0.90 (0.85 to 0.96)	<.001	0.89 (0.84 to 0.94)	<.001	0.90 (0.85 to 0.96)	.001
X-ray imaging^a^	38 093 (81.4)	100 751 (80.7)	0.96 (0.91 to 1.01)	.10	0.94 (0.89 to 0.99)	.02	0.94 (0.89 to 0.99)	.02
Endoscopy^a^^,^^b^	4247 (9.1)	11 340 (9.1)	0.96 (0.91 to 1.01)	.12	0.95 (0.90 to 1.01)	.09	0.96 (0.90 to 1.01)	.14
Routine blood tests per day, median (IQR)^c^^,^^d^	3.20 (2.23 to 4.38)	3.23 (2.20 to 4.43)	0.99 (0.97 to 1.01)	.14	0.98 (0.96 to 1.00)	.05	0.99 (0.98 to 1.01)	.37
Acute blood tests^a^^,^^e^	34 935 (74.7)	91 245 (73.1)	1.02 (0.96 to 1.08)	.54	1.01 (0.94 to 1.07)	.84	1.00 (0.94 to 1.06)	.88
Blood transfusion^a^	2939 (6.3)	8162 (6.5)	0.96 (0.90 to 1.03)	.27	0.99 (0.91 to 1.07)	.74	0.98 (0.91 to 1.06)	.67
Ultrasound^a^	14 832 (31.7)	36 195 (29.0)	0.93 (0.87 to 1.00)	.05	0.91 (0.85 to 0.97)	.006	0.91 (0.85 to 0.97)	.004
Interventional radiology^a^	3692 (7.9)	9831 (7.9)	0.96 (0.90 to 1.03)	.29	0.97 (0.90 to 1.04)	.32	0.96 (0.90 to 1.02)	.21
Antipsychotics^a^	8731 (18.7)	22 439 (18.0)	1.00 (0.96 to 1.05)	.97	1.00 (0.96 to 1.04)	.88	0.99 (0.96 to 1.04)	.77
Antimicrobials^a^	24 578 (52.5)	63 770 (51.1)	0.97 (0.93 to 1.00)	.07	0.96 (0.92 to 1.00)	.04	0.97 (0.93 to 1.01)	.10
Benzodiazepines^a^	12 913 (27.6)	32 752 (26.2)	0.98 (0.94 to 1.03)	.45	0.97 (0.93 to 1.01)	.16	0.97 (0.93 to 1.00)	.08
Anticoagulants^a^	7533 (16.1)	19 638 (15.7)	0.98 (0.95 to 1.02)	.35	1.01 (0.97 to 1.04)	.79	1.02 (0.98 to 1.05)	.36

Abbreviations: CT, computed tomography; IQR, interquartile range; MRI, magnetic resonance imaging; OR, odds ratio; RR, rate ratio.

^a^ Odds ratio from logistic regression.

^b^ Endoscopy included sigmoidoscopy, colonoscopy, esophagogastroduodenoscopy, endoscopic retrograde cholangiopancreatography and/or bronchoscopy.

^c^ Rate ratio from negative binomial regression.

^d^ Complete blood cell count and/or electrolytes.

^e^ Lactate, troponin, and/or blood gases.

### Sensitivity Analyses

When we included only those hospitalizations for which the MRP was also the attending and discharging physician, patients of female physicians had significantly lower mortality, which persisted in all adjusted models (AOR, 1.10; 95% CI, 1.01-1.19; *P* = .03) (eTable 3 in the Supplement). Processes of care findings were similar to the main model (eTable 4 in Supplement). In a second sensitivity analysis, we excluded palliative encounters (eTables 5 and 6 in the Supplement). Mortality decreased by greater than 50%, and the unadjusted difference between female and male physicians was significant (AOR, 1.09; 95% CI, 1.01-1.18; *P* = .02) and was attenuated after adjustment for physician characteristics, similar to the main model. Estimates of the association of physician gender in unadjusted models and models adjusting for patient characteristics were similar when limited only to male or female patients, and these associations were attenuated after adjustment for physician characteristics (eTable 7 in the Supplement). Models additionally adjusting for patient characteristics and physician years of experience demonstrated attenuation of the physician gender association as well (eTable 8 in the Supplement). This attenuation persisted whether years of experience was added as a linear term, categorical term, or quadratic and cubic terms (eTable 9 in the Supplement).

## Discussion

In a Canadian population, we observed that hospitalized general medicine patients of female physicians had lower in-hospital mortality rates compared with their male counterparts. Specifically, the in-hospital mortality rate was 0.47% lower for patients of female physicians in the cohort after adjustment for hospital effects and patient characteristics. This finding is similar to the 0.43% adjusted 30-day mortality difference noted by Tsugawa et al^1^ in a US Medicare population. Similar to the study by Tsugawa and colleagues,^1^ we found that male physicians had more years of experience than female physicians and that adjusting for this variable and other physician characteristics attenuated the mortality difference associated with physician gender.

We were able to account for many processes of care: routine and acute blood tests, invasive and noninvasive diagnostic imaging tests, and medications ordered by the MRP. We hypothesized that physician gender–based differences in care may explain a difference in patient mortality. The limited literature on physician gender-mediated differences in care suggests that female physicians may spend more time reading electronic health records^50^ and may prescribe certain medications with additional caution.^51,52,53^ Furthermore, evidence from previous studies suggests that female physicians perceive clinical risks more highly^54,55^ and, perhaps as a result, order more tests^56,57^ and request more referrals^57,58^ than their male counterparts. In line with this hypothesis, we found that female physicians ordered more diagnostic CT, MRI, and ultrasound imaging tests than male physicians. However, the frequency and type of diagnostic tests ordered by physicians did not attenuate the difference in mortality rate. These findings raise the question: what drives the lower mortality rate in patients of female physicians?

The in-hospital mortality difference between patients of female and male physicians was attenuated after adjustment for other physician characteristics. Physician years of experience was the only physician characteristic in our model that differed significantly between male and female physicians, and greater years of experience was independently associated with increased patient mortality. Some suggest that physicians closer to their residency training are more up to date on clinical guidelines and more likely to follow evidence-based practice, which may improve patient outcomes.^59^ Recent studies in internal medicine reported that a longer period of time since medical school graduation^60^ and older physician age^61^ were significantly associated with increased patient mortality. This association may be diminished when physicians treat higher volumes of patients.^60,61^ The growing proportion of female physicians entering the Canadian workforce^62^ may help to explain our finding that the mortality difference was attenuated after adjusting for other physician characteristics.

Gender-mediated behavioral differences that are difficult to measure through routinely collected electronic data may also play a role in explaining the mortality difference. Some studies have shown than female physicians are more likely than male physicians to provide patient-centered care,^63^ spend longer communicating with their patients,^64^ provide more nonverbal feedback,^65^ and show higher levels of empathic concern.^66,67^ Humanistic relationships with patients may enable increased patient disclosure of medical information^65,68^ and foster stronger relationships among health team members, thereby improving patient care. Furthermore, female physicians, on average,^51,52,53,54^ may obtain more frequent informal consultations with colleagues and be more focused on reading clinical research studies or reviewing a patient’s chart when making clinical decisions. Taken together, these differences in process may help to explain the modestly lower mortality rates among general medical patients treated by female physicians in ways that cannot be captured through electronic health records or administrative data.

The results of this study raise pertinent questions regarding the factors contributing to physician gender-mediated differences in processes of care and patient outcomes. In interpreting these findings, we exercise caution to avoid perpetuating gender stereotypes. Female and male physicians may have been socialized to adhere to gender norms and expectations within a health care context,^69,70^ but such behavioral differences are modifiable and not fixed.^71^

### Limitations

Our study had several limitations. First, we were restricted to reporting in-hospital deaths as opposed to 30-day mortality. Second, the 30-day readmission rate only accounted for readmissions to the 7 hospital sites included. In the greater Toronto area, 82% of readmissions are estimated to occur at the same hospital^72^; because our study included patients readmitted to 6 other local hospitals, our coverage of all readmissions was probably higher. Third, the designation of the MRP for each patient was an approximation, as it was common for more than 1 physician to be involved in the care of complex hospitalized patients. Our assumption that 1 physician provided most of the care may have either minimized or exaggerated our findings. We sought to reduce the possibility of transitions between physicians by only including patients whose total LOS was less than 30 days. In a sensitivity analysis in which the MRP, admitting physician, and discharging physician were the same, thereby limiting the possibility of patient handover, the mortality difference persisted (eTable 2 in the Supplement).

Another limitation of our study was that we could not define physician gender beyond a binary framing of female and male. Furthermore, we could not include other relevant physician characteristics, such as race/ethnicity, religion, sexual orientation, and country of origin, because these variables were unavailable in the CPSO physician database. Using a more intersectional lens would better capture the complexities of physician identity and its role in patient care.^73^

This study was also limited in its generalizability, as the care was provided by 172 physicians in 1 region in Canada. Although these data may not be representative of Canadian hospitals at large, our analysis did include GIM and hospitalist physicians working at both academic and community hospitals in urban and suburban areas.

## Conclusions

This multisite, retrospective, cross-sectional study assessed the association of physician gender with processes of care and outcomes of patients hospitalized in Canadian GIM wards. Patients of female physicians had lower mortality than those of male physicians when adjusted for hospital and patient characteristics. However, this difference was nonsignificant after adjustment for other physician characteristics including age, years of experience, and location of medical school training. Future research should seek to validate these findings and explore additional processes of care and behaviors of physicians that may explain differences in patient mortality associated with physician gender.

## Notes

Supplement. **eTable 1.** Changes in Proportion of Patients Seen by Female vs Male Physicians Over Time **eTable 2.** Additional Models: Outcomes in Models Adjusting for Hospital Effects, Patient Characteristics and Processes of Care Only **eTable 3.** Sensitivity Analysis 1: Outcomes From Cohort Restricted to Patients With the Same Most Responsible, Admitting and Discharging Physicians **eTable 4.** Sensitivity Analysis 1: Processes of Care From Cohort Restricted to Patients With the Same Most Responsible, Admitting and Discharging Physicians **eTable 5.** Sensitivity Analysis 2: Outcomes From Cohort Restricted to Nonpalliative Admissions **eTable 6.** Sensitivity Analysis 2: Univariable and Multivariable Processes of Care From Cohort Restricted to Nonpalliative Admissions **eTable 7.** Sensitivity Analysis 3: In-Hospital Mortality Among Cohorts of Male-Only and Female-Only Patients **eTable 8.** Sensitivity Analysis 4: Outcomes in Models Including Physician Experience Only **eTable 9.** Sensitivity Analysis 5: Models of In-Hospital Mortality With Years of Experience Included in Different Forms **eTable 10.** Medication Variable Mapping
