# Supplementary material for: Variations in Processes of Care and Outcomes for Hospitalized General Medicine Patients Treated by Female vs Male Physicians
Source: JAMA Health Forum. 2021 Jul 16;2(7):e211615. doi: 10.1001/jamahealthforum.2021.1615 (PMC8796959; doi:10.1001/jamahealthforum.2021.1615)
Supplement: Supplement. — eTable 1. Changes in Proportion of Patients Seen by Female vs Male Physicians Over Time eTable 2. Additional Models: Outcomes in Models Adjusting for Hospital Effects, Patient Characteristics and Processes of Care Only eTable 3. Sensitivity Analysis 1: Outcomes From Cohort Restricted to Patients With the Same Most Responsible, Admitting and Discharging Physicians eTable 4. Sensitivity Analysis 1: Processes of Care From Cohort Restricted to Patients With the Same Most Responsible, Admitting and Discharging Physicians eTable 5. Sensitivity Analysis 2: Outcomes From Cohort Restricted to Nonpalliative Admissions eTable 6. Sensitivity Analysis 2: Univariable and Multivariable Processes of Care From Cohort Restricted to Nonpalliative Admissions eTable 7. Sensitivity Analysis 3: In-Hospital Mortality Among Cohorts of Male-Only and Female-Only Patients eTable 8. Sensitivity Analysis 4: Outcomes in Models Including Physician Experience Only eTable 9. Sensitivity Analysis 5: Models of In-Hospital Mortality With Years of Experience Included in Different Forms eTable 10. Medication Variable Mapping [file jamahealthforum-e211615-s001.pdf]

Supplementary Online Content

Sergeant A, Saha S, Shin S, et al. Variations in processes of care and outcomes for hospitalized general medicine patients treated by female vs male physicians. *JAMA Health Forum*. 2021;2(7):e211615. doi:10.1001/jamahealthforum.2021.1615

eTable 1. Changes in Proportion of Patients Seen by Female vs Male Physicians Over Time

eTable 2. Additional Models: Outcomes in Models Adjusting for Hospital Effects, Patient Characteristics and Processes of Care Only

eTable 3. Sensitivity Analysis 1: Outcomes From Cohort Restricted to Patients With the Same Most Responsible, Admitting and Discharging Physicians

eTable 4. Sensitivity Analysis 1: Processes of Care From Cohort Restricted to Patients With the Same Most Responsible, Admitting and Discharging Physicians

eTable 5. Sensitivity Analysis 2: Outcomes From Cohort Restricted to Nonpalliative Admissions

eTable 6. Sensitivity Analysis 2: Univariable and Multivariable Processes of Care From Cohort Restricted to Nonpalliative Admissions

eTable 7. Sensitivity Analysis 3: In-Hospital Mortality Among Cohorts of Male-Only and Female-Only Patients

eTable 8. Sensitivity Analysis 4: Outcomes in Models Including Physician Experience Only

eTable 9. Sensitivity Analysis 5: Models of In-Hospital Mortality With Years of Experience Included in Different Forms

eTable 10. Medication Variable Mapping

This supplementary material has been provided by the authors to give readers additional information about their work.

© 2021 Sergeant A et al. *JAMA Health Forum.*

eTable 1. Changes in Proportion of Patients Seen by Female vs Male Physicians Over Time

Fiscal year of Patient Admission (%)	Proportion of Female Physician Admissions	Female Physicians	Male Physicians	P-value	Standardized Mean Difference
2010	20.4%	3285 (7.0)	12790 (10.2)	<0.001	0.210
2011	21.2%	4494 (9.6)	16689 (13.4)		
2012	24.4%	5454 (11.7)	16941 (13.6)		
2013	27.1%	6315 (13.5)	17012 (13.6)		
2014	30.2%	7436 (15.9)	17172 (13.8)		
2015	32.0%	8016 (17.1)	17042 (13.6)		
2016	30.8%	7709 (16.5)	17295 (13.9)		
2017	29.0%	4063 (8.7)	9912 (7.9)		

© 2021 Sergeant A et al. *JAMA Health Forum.*

eTable 2. Additional Models: Outcomes in Models Adjusting for Hospital Effects, Patient Characteristics and Processes of Care Only

	**Main Cohort**		**Sensitivity 1 Cohort (admissions with same admitting, most-responsible and discharging physician)**		**Sensitivity 2 Cohort (non-palliative admissions)**	
**Outcome**	OR / RR / Effect (CI)	p-val	OR / RR / Effect (CI)	p-val	OR / RR / Effect (CI)	p-value
Mortality[a]	1.13 (1.03, 1.24)	0.013	1.12 (1.03, 1.21)	0.005	1.11 (1.01, 1.23)	0.029
30-day Readmission[a]	1.02 (0.98, 1.06)	0.389	1.04 (0.99, 1.09)	0.172	1.02 (0.98, 1.06)	0.401
ICU Admission[a]	1.00 (0.92, 1.09)	0.978	0.97 (0.87, 1.08)	0.540	0.99 (0.91, 1.09)	0.879
Length of Stay[b]	0.97 (0.96, 0.99)	0.001	0.99 (0.97, 1.01)	0.343	0.97 (0.96, 0.99)	0.001
Total Cost[c]	-3.64% (-5.26%, -2.00%)	<0.001	-2.88% (-4.87%, -0.84%)	0.006	-3.68% (-5.35%, -1.97%)	<0.001

© 2021 Sergeant A et al. *JAMA Health Forum.*

eTable 3. Sensitivity Analysis 1: Outcomes From Cohort Restricted to Patients With the Same Most Responsible, Admitting and Discharging Physicians

	**Model 1** (hospital effects)		**Model 2** (+ patient characteristics)		**Model 3** (+physician characteristics)		**Model 4** (+ processes of care)	
**Outcome**	OR / RR / Effect (CI)	p-val	OR / RR / Effect (CI)	p-val	OR / RR / Effect (CI)	p-val	OR /RR / Effect (CI)	p-val
Mortality[a]	1.12 (1.04, 1.20)	0.003	1.10 (1.02, 1.19)	0.013	1.09 (1.00, 1.18)	0.042	1.10 (1.01, 1.19)	0.025
30-day Readmission[a]	1.03 (0.98, 1.09)	0.221	1.04 (0.99, 1.09)	0.151	1.04 (1.00, 1.09)	0.075	1.04 (1.00, 1.09)	0.078
ICU Admission[a]	0.92 (0.83, 1.03)	0.152	0.92 (0.83, 1.03)	0.140	0.92 (0.82, 1.02)	0.121	0.95 (0.86, 1.06)	0.378
Length of Stay[b]	1.01 (0.97, 1.06)	0.518	1.00 (0.96, 1.04)	0.930	0.99 (0.96, 1.03)	0.591	0.99 (0.97, 1.01)	0.301
Total Cost[c]	-1.92% (-5.70%, 2.00%)	0.332	-2.89% (-6.21%, 0.56%)	0.100	-3.41% (-6.61%, -0.10%)	0.044	-2.74% (-4.80%, -0.64%)	0.011

a. Odds Ratio (OR) from logistic regression
b. Rate Ratio (RR) (days per admission) from negative binomial regression
c. Effect Size on log of total cost expressed as percentage change

© 2021 Sergeant A et al. *JAMA Health Forum.*

eTable 4. Sensitivity Analysis 1: Processes of Care From Cohort Restricted to Patients With the Same Most Responsible, Admitting and Discharging Physicians

	**Model 1** (hospital effects)		**Model 2** (+ patient characteristics)		**Model 3** (+ physician characteristics)	
**Process of Care**	OR / RR / Effect (CI)	p-val	OR / RR / Effect (CI)	p-val	OR / RR / Effect (CI)	p-val
CT Imaging[a]	0.92 (0.88, 0.98)	0.004	0.91 (0.86, 0.97)	0.002	0.92 (0.87, 0.98)	0.009
MRI Imaging[a]	0.91 (0.84, 0.98)	0.019	0.89 (0.82, 0.96)	0.003	0.89 (0.82, 0.97)	0.007
X-Ray Imaging[a]	0.98 (0.92, 1.03)	0.379	0.95 (0.90, 1.00)	0.051	0.95 (0.90, 1.01)	0.094
Endoscopy[a]	0.97 (0.91, 1.03)	0.322	0.96 (0.89, 1.03)	0.270	0.96 (0.89, 1.04)	0.267
Routine Bloodwork Per Day[b]	0.97 (0.95, 0.99)	0.006	0.97 (0.95,0.99)	0.003	0.98 (0.96, 1)	0.049
Acute Bloodwork[a]	1.04 (0.97, 1.11)	0.295	1.02 (0.95, 1.10)	0.593	1.02 (0.95, 1.10)	0.566
Blood Transfusion[a]	1.01 (0.92, 1.11)	0.884	1.02 (0.92, 1.13)	0.670	1.02 (0.92, 1.12)	0.762
Ultrasound[a]	0.95 (0.87, 1.02)	0.164	0.92 (0.85, 1.00)	0.052	0.92 (0.85, 0.99)	0.033
Interventional Radiology[a]	1.01 (0.91, 1.11)	0.924	0.99 (0.90, 1.10)	0.914	0.99 (0.90, 1.08)	0.803
Antipsychotics[a]	0.99 (0.94, 1.04)	0.646	0.97 (0.93, 1.02)	0.224	0.97 (0.93, 1.02)	0.237
Antimicrobials[a]	0.98 (0.93, 1.02)	0.271	0.97 (0.93, 1.02)	0.235	0.98 (0.93, 1.03)	0.376
Benzodiazepines[a]	0.98 (0.92, 1.03)	0.378	0.96 (0.91, 1.02)	0.192	0.96 (0.91, 1.01)	0.099
Anticoagulants[a]	1.01 (0.97, 1.05)	0.748	1.03 (0.99, 1.07)	0.223	1.03 (0.99, 1.08)	0.103

a. Odds Ratio (OR) from logistic regression
b. Rate Ratio (RR) (days per admission) from negative binomial regression

© 2021 Sergeant A et al. *JAMA Health Forum.*

eTable 5. Sensitivity Analysis 2: Outcomes From Cohort Restricted to Nonpalliative Admissions

	**Model 1** (+ hospital effects)		**Model 2** (+ patient characteristics)		**Model 3** (+ physician characteristics)		**Model 4** (+ processes of care)	
**Outcome**	OR / RR / Effect (CI)	p-val	OR / RR / Effect (CI)	p-val	OR / RR / Effect (CI)	p-value	OR / RR / Effect (CI)	p-val
Mortality[a]	1.09 (1.01, 1.18)	0.021	1.09 (0.99, 1.21)	0.075	1.06 (0.96, 1.18)	0.254	1.06 (0.95, 1.17)	0.296
30-day Readmission[a]	1.01 (0.97, 1.05)	0.679	1.02 (0.98, 1.06)	0.394	1.03 (0.99, 1.07)	0.218	1.03 (0.99, 1.07)	0.195
ICU Admission[a]	0.95 (0.87, 1.04)	0.281	0.96 (0.87, 1.05)	0.337	0.92 (0.85, 1.00)	0.047	0.94 (0.86, 1.03)	0.179
Length of Stay[b]	0.97 (0.94, 1.00)	0.034	0.97 (0.94, 0.99)	0.018	0.97 (0.94, 0.99)	0.009	0.97 (0.96, 0.99)	0.006
Total Cost[c]	-5.22% (-8.42%, -1.91%)	0.002	-5.09% (-7.92%, -2.18%)	0.001	-5.34% (-8.04%, -2.55%)	<0.001	-3.48% (-5.19%, -1.74%)	<0.001

a. Odds Ratio (OR) from logistic regression
b. Rate Ratio (RR) (days per admission) from negative binomial regression
c. Effect Size on log of total cost expressed as percentage change

© 2021 Sergeant A et al. *JAMA Health Forum.*

eTable 6. Sensitivity Analysis 2: Univariable and Multivariable Processes of Care From Cohort Restricted to Nonpalliative Admissions

	**Model 1** (+ hospital effects)		**Model 2** (+ patient characteristics)		**Model 3** (+ physician characteristics)	
**Process of Care**	OR / RR / Effect (CI)	p-val	OR / RR / Effect (CI)	p-val	OR / RR / Effect (CI)	p-val
CT Imaging[a]	0.91 (0.88, 0.95)	<0.001	0.91 (0.87, 0.96)	<0.001	0.93 (0.89, 0.98)	0.004
MRI Imaging[a]	0.91 (0.86, 0.96)	0.001	0.89 (0.84, 0.95)	<0.001	0.90 (0.85, 0.96)	0.001
X-Ray Imaging[a]	0.95 (0.90, 1.00)	0.063	0.93 (0.88, 0.99)	0.016	0.93 (0.88, 0.99)	0.014
Endoscopy[a]	0.96 (0.91, 1.01)	0.128	0.95 (0.90, 1.01)	0.111	0.96 (0.90, 1.02)	0.149
Routine Bloodwork Per Day[b]	0.99 (0.97, 1.01)	0.228	0.99 (0.97, 1.00)	0.108	0.99 (0.98, 1.01)	0.491
Acute Bloodwork[a]	1.02 (0.96, 1.08)	0.522	1.01 (0.95, 1.08)	0.776	1.00 (0.94, 1.06)	0.933
Blood Transfusion[a]	0.95 (0.89, 1.02)	0.185	0.98 (0.91, 1.06)	0.666	0.98 (0.91, 1.06)	0.620
Ultrasound[a]	0.93 (0.86, 1.00)	0.035	0.90 (0.84, 0.97)	0.005	0.90 (0.84, 0.97)	0.003
Interventional Radiology[a]	0.97 (0.91, 1.04)	0.439	0.98 (0.91, 1.05)	0.503	0.97 (0.90, 1.03)	0.308
Antipsychotics[a]	1.00 (0.96, 1.04)	0.939	0.99 (0.96, 1.03)	0.732	1.00 (0.96, 1.04)	0.967
Antimicrobials[a]	0.96 (0.93, 1.00)	0.063	0.96 (0.92, 1.00)	0.041	0.97 (0.93, 1.01)	0.116
Benzodiazepines[a]	0.98 (0.94, 1.02)	0.236	0.96 (0.92, 1.00)	0.045	0.96 (0.92, 1.00)	0.046
Anticoagulants[a]	0.98 (0.94, 1.02)	0.340	1.01 (0.97, 1.04)	0.724	1.02 (0.98, 1.05)	0.373

a. Odds Ratio
b. Effect Size

© 2021 Sergeant A et al. *JAMA Health Forum.*

eTable 7. Sensitivity Analysis 3: In-Hospital Mortality Among Cohorts of Male-Only and Female-Only Patients

	**Model 1** (+ hospital effects)		**Model 2** (+ patient characteristics)		**Model 3** (+ physician characteristics)		**Model 4** (+ processes of care)	
**Cohort**	OR (CI)	p-val	OR (CI)	p-val	OR (CI)	p-val	OR (CI)	p-val
Male patients only	1.11 (0.99, 1.23)	0.070	1.12 (1.00, 1.25)	0.055	1.08 (0.98, 1.21)	0.137	1.10 (0.98, 1.22)	0.097
Female patients only	1.12 (0.99, 1.25)	0.053	1.12 (0.99, 1.26)	0.070	1.05 (0.95, 1.17)	0.330	1.05 (0.94, 1.17)	0.425

© 2021 Sergeant A et al. *JAMA Health Forum.*

eTable 8. Sensitivity Analysis 4: Outcomes in Models Including Physician Experience Only

	**Model including hospital fixed effects, patient characteristics and physician years of experience**	
**Outcome**	OR / RR / Effect (CI)	p-val
Mortality[a]	1.07 (0.98, 1.17)	0.122
30-day Readmission[a]	1.02 (0.98, 1.07)	0.244
ICU Admission[a]	0.94 (0.86, 1.01)	0.107
Length of Stay[b]	0.97 (0.94, 0.99)	0.017
Total Cost[c]	-5.06% (-7.76%, -2.28%)	<0.001

a. Odds Ratio (OR) from logistic regression
b. Rate Ratio (RR) (days per admission) from negative binomial regression
c. Effect Size on log of total cost expressed as percentage change

© 2021 Sergeant A et al. *JAMA Health Forum.*

eTable 9. Sensitivity Analysis 5: Models of In-Hospital Mortality With Years of Experience Included in Different Forms

	**Model 1** (+ hospital effects)		**Model 2** (+ patient characteristics)		**Model 3** (+ physician characteristics)		**Model 4** (+ processes of care)	
**Cohort**	OR (CI)	p-val	OR (CI)	p-val	OR (CI)	p-val	OR (CI)	p-val
Linear (Main Analysis)	1.11 (1.01, 1.23)	0.036	1.12 (1.01, 1.24)	0.032	1.07 (0.98, 1.17)	0.124	1.07 (0.99, 1.17)	0.104
Categorical	1.11 (1.01, 1.23)	0.036	1.12 (1.01, 1.24)	0.032	1.07 (0.99, 1.16)	0.099	1.07 (1.00, 1.17)	0.085
Quadratic and Cubic Terms	1.11 (1.01, 1.23)	0.036	1.118 (1.01, 1.24)	0.032	1.06 (0.98, 1.15)	0.134	1.07 (0.99, 1.16)	0.114

© 2021 Sergeant A et al. *JAMA Health Forum.*

eTable 10. Medication Variable Mapping

Medication Variable	Definition
Antimicrobials	antibiotics, antifungals and antivirals, except TB-specific medications, anti-retrovirals, and any topical and ophthalmic preparations
Anticoagulants	warfarin, acenocoumarol, apixaban, argatroban, bivalirudin, dabigatran, desirudin, edoxaban, lepirudin, melagatran, rivaroxaban, ximelagatran
Anti-psychotics	aripiprazole, asenapine, chlorpromazine, clozapine, droperidol, flupentixol, fluphenazine, haloperidol, lurasidone, loxapine, methotrimeprazine, olanzapine, paliperidone, perphenazine, prochlorperazine, prochlorazine, pimozide, pipotiazine, quetiapine, risperidone, sulpiride, trifluoperazine, urasidone, zuclopenthixol, ziprasidone
Benzodiazepines	"-pams ", clobazam, chlordiazapoxide, clorazepate, diazepam rectal gel, fluorazapam

© 2021 Sergeant A et al. *JAMA Health Forum.*
